# Conservative treatment of a left atrial intramural hematoma after left atrial thrombus resection and concomitant mitral valve replacement - case report

**DOI:** 10.1186/1749-8090-6-50

**Published:** 2011-04-13

**Authors:** Dirk Bruegger, Sebastian Sadoni, Mikhail Primaychenko, Ralf Sodian, Christoph Schmitz, Bruno Reichart, Daniel Chappell

**Affiliations:** 1 Department of Anesthesiology, Ludwig-Maximilians-University Munich, Marchioninistrasse 15, 81377 Munich, Germany; 2 Department of Cardiac Surgery, Ludwig-Maximilians-University Munich, Marchioninistrasse 15, 81377 Munich, Germany

## Abstract

Left atrial intramural hematoma is a seldom cause of left atrial mass. It has been described to occur spontaneously, after interventional procedures, after blunt chest trauma, or after aortocoronary bypass surgery. We present a case of mitral valve replacement together with the removal of a large intraatrial space-occupying lesion. Intraoperative transesophageal echocardiography confirmed a successful resection of this mass. Surprisingly, upon admission to ICU, transesophageal and transthoracic echocardiography revealed a recurrence of an intramural lesion, closest matching a hematoma, which was confirmed by contrast-enhanced computed tomography. Surgical intervention was thoroughly discussed but a conservative management was favoured. 3 months after surgery, a reassessed transthoracic echocardiography and computed tomography demonstrated an almost complete resolution of the pre-existing hematoma.

## Background

Atrial intramural hematomas are severe but rare complications of cardiac surgery and only few cases are described in literature. We present a case of an unexpected intramural left atrial hematoma following mitral valve replacement and concomitant left atrial thrombus resection and the beneficial role of perioperative echocardiography in detecting and monitoring this event.

## Case presentation

**Figure 1:**
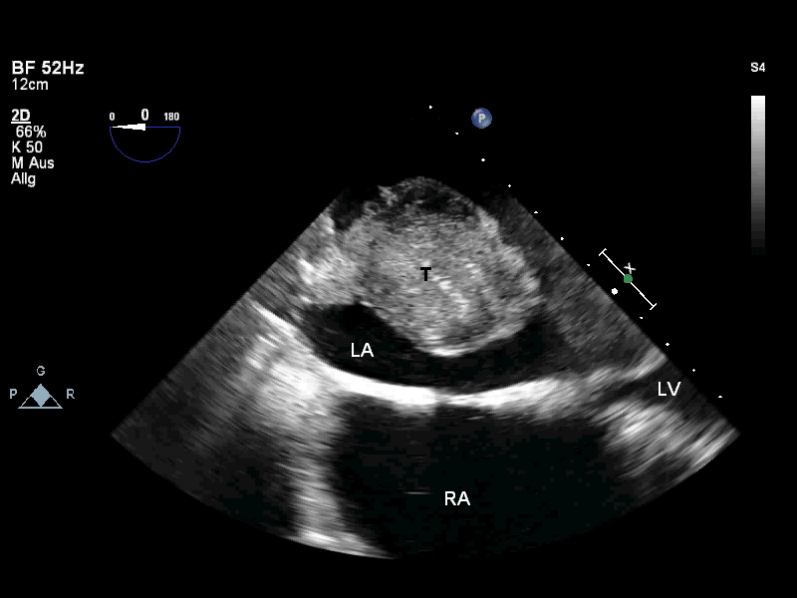
**Preoperative transesophageal echocardiogram demonstrating a voluminous intracavitary mass (58 × 45 mm) attached to the left atrial roof**. T: thrombus; LA: left atrium; LV: left ventricle; RA: right atrium.

A 76-year-old woman with a history of intermittent atrial fibrillation and cerebral infarction with left-sided hemiparesis was admitted to our hospital. Preoperative transesophageal echocardiography revealed a dilated left atrium (90 × 80 mm), mitral valve stenosis (mitral valve area 1.04 cm^2^, transmitral pressure gradient 9 mmHg), and the presence of a large intracavitary, space-occupying lesion attached to the left atrial roof (Figure 1, Additional file 1).

The patient received mitral valve replacement via left atriotomy with a biological prothesis (Perimount magna 27 mm; Edwards Lifesciences, Irvine, CA, USA), extirpation of the intracavitary mass and ligation of the left atrial appendage. Intraoperative transesophageal echocardiography revealed satisfactory valve function and confirmed the successful removal of the intracavitary lesion. Histopathological examination revealed a spherical mass which was subsequently confirmed to be a partially calcified and connective tissue-organized thrombus.

**Figure 2:**
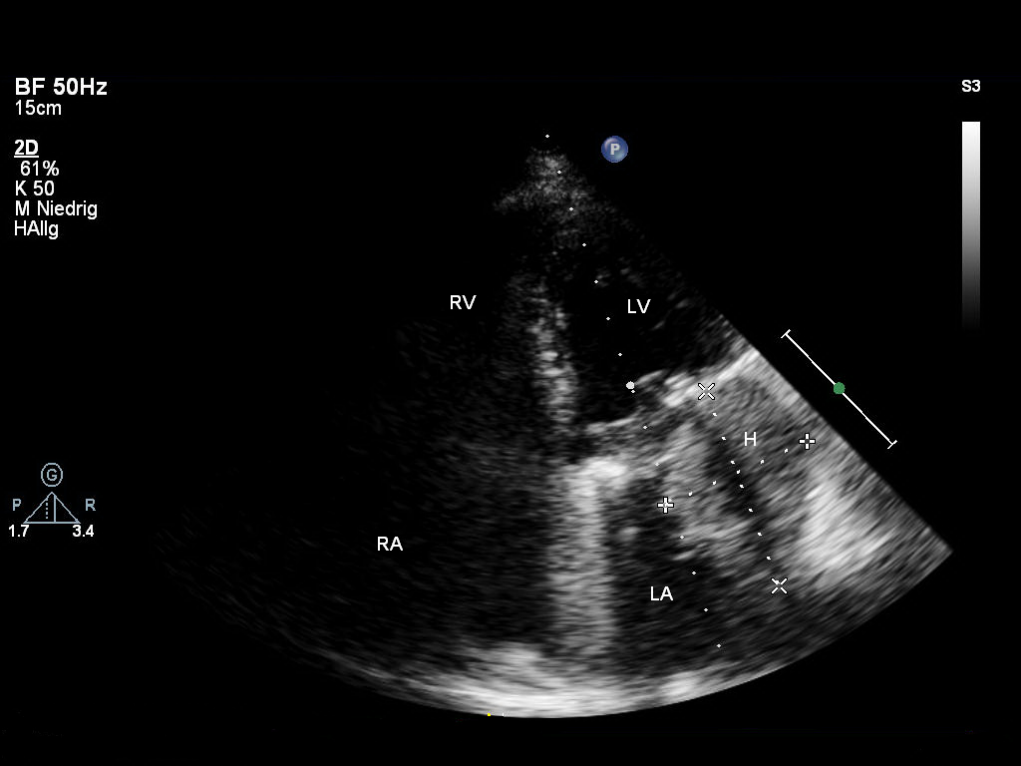
**Postoperative transthoracic echocardiogram, apical 4-chamber view, showing development of an echogenic mass (54 × 40 mm) compatible with an intramural left atrial hematoma**. RV: right ventricle; LV: left ventricle; RA: right atrium; LA: left atrium; H: intramural hematoma.

**Figure 3:**
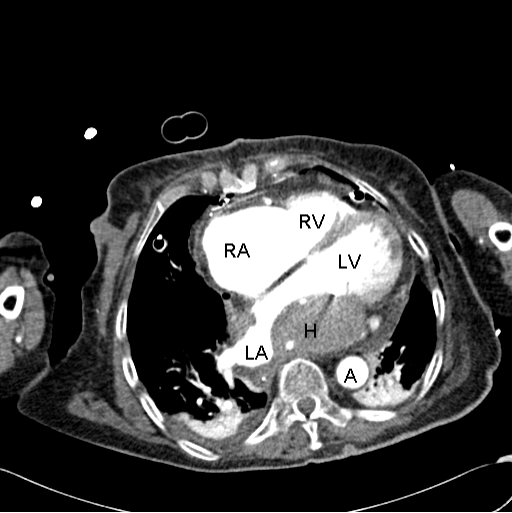
**Postoperative contrast-enhanced computed tomography scan confirming development of a large intramural hematoma almost obliterating the left atrial chamber**. RA: right atrium; RV: right ventricle; LA: left atrium; LV: left ventricle; H: intramural hematoma; A: descending aorta.

Surprisingly, upon admission to ICU echocardiography revealed a reappearance of a new homogenous mass arising from the left atrial wall (Figure 2, Additional file 2). Neither cardiac output nor ejection fraction were significantly influenced by the hematoma. Contrast-enhanced computed tomography was performed and showed a hyperdense, even bordered, intramural hematoma in the left dorso-basal atrium subtotally obstructing the left atrial chamber (Figure 3, Additional file 3). Surgical intervention was thoroughly discussed interdisciplinary but in the light of the patient's hemodynamic stability and the high risk of dissection of the atrial wall at the auriculoventricular annulus, the risk-benefit analysis favoured a conservative management. Therapeutic dose of intravenous unfractionated heparin was started without a bolus 24 hours after surgery with a target PTT value of 50 seconds. Heparin was continued until an oral anticoagulation therapy with warfarin was in the target INR range of 2.0 to 3.0.

**Figure 4:**
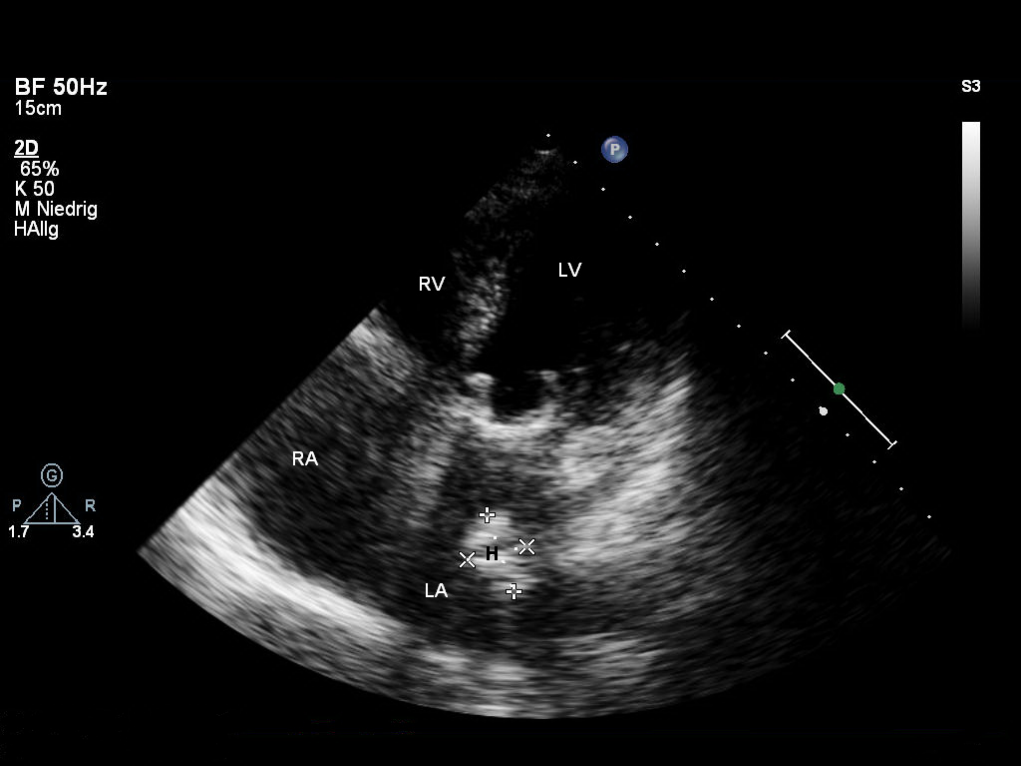
**Transthoracic echocardiogram with apical 4-chamber view performed three months following surgery showing a small residual hematoma (21 × 16 mm) in the left atrium**. RV: right ventricle; LV: left ventricle; RA: right atrium; LA: left atrium; H: intramural hematoma.

**Figure 5:**
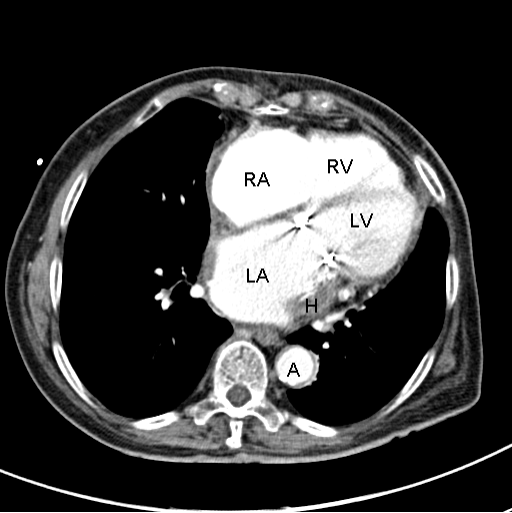
**Contrast-enhanced computed tomography scan three months following surgery revealing an almost complete absorption of the intramural hematoma**. RA: right atrium; RV: right ventricle; LA: left atrium; LV: left ventricle; H: intramural hematoma; A: descending aorta.

In-house follow up serial echocardiography demonstrated a stable-to-improving left atrial hematoma. A transthoracic echocardiography and contrast-enhanced computed tomography 3 months after surgery revealed an almost complete resolution of the pre-existing hematoma (Figures 4 and 5, Additional files 4 and 5).

## Discussion

Intramural left atrial hematomas are a very uncommon entity, but have been described to occur spontaneously [1-4], after aortocoronary bypass surgery [5], after percutaneous coronary interventions [6,7], radiofrequency catheter ablations [8,9] and after blunt chest trauma [10].

In our case, routine postoperative transesophageal echocardiography revealed an acute 5 cm diameter intramural hematoma in the left atrium, which could have remained undetected as our patient was asymptomatic. Pre- [11] and intraoperative [12] echocardiography of the heart is an indispensible examination for patients undergoing cardiac valve surgery. Whereas transthoracal echocardiography is less invasive and more convenient to the patient, several studies have demonstrated transesophageal echocardiography to be superior for assessing possible sources of cardiac embolism [13], such as intracardiac thrombi or intramural lesions [14]. As in our case, latter can ensure a quick and accurate diagnosis in combination with a computed tomography.

Judging by the spontaneous regression, a conservative approach seemed justified, despite anticoagulation.

## Conclusion

A conservative approach with close-meshed serial echocardiographic examinations in a hemodynamically stable and asymptomatic patient with a left atrial intramural hematoma seems to be an appropriate strategy. This case demonstrates the usefulness and necessity of perioperative echocardiographic imaging in the detection and monitoring of this unexpected event.

## Consent

Written informed consent was obtained from the patient for publication of this case report and any accompanying images. A copy of the written consent is available for review by the Editor-in-Chief of this journal.

## Competing interests

The authors declare that they have no competing interests.

## Authors' contributions

DB reviewed the case, conducted a review of the literature and drafted the manuscript. SS and MP performed the echocardiographic studies and participated in the design of the case report. RS and CS performed the operation described. BR and DC confirmed the patient's diagnosis and revised the manuscript, contributing important intellectual content. All authors read and approved the final manuscript.

## Supplementary Material

Additional file 1 **Preoperative transesophageal echocardiogram demonstrating a voluminous intracavitary mass attached to the left atrial roof**. Click here for file

Additional file 2 **Postoperative transthoracic echocardiogram showing development of an echogenic mass arising from the left atrial wall**. Click here for file

Additional file 3 **Postoperative axial CT images**. Click here for file

Additional file 4 **Transthoracic echocardiogram three months following surgery revealing an almost complete resolution of the pre-existing hematoma**. Click here for file

Additional file 5 **Axial CT images three months following surgery**. Click here for file
